# Framework for multi-stressor physiological response evaluation in amphibian risk assessment and conservation

**DOI:** 10.3389/fevo.2024.1336747

**Published:** 2024-03-13

**Authors:** Jill A. Awkerman, Donna A. Glinski, W. Matthew Henderson, Robin Van Meter, S. Thomas Purucker

**Affiliations:** 1 Center for Ecosystem Measurement and Modeling, Office of Research and Development, US Environmental Protection Agency, Gulf Breeze, FL, United States; 2 Center for Ecosystem Measurement and Modeling, Office of Research and Development, US Environmental Protection Agency, Athens, GA, United States; 3 Environmental Science and Studies, Washington College, Chestertown, MD, United States; 4 Center for Computational Toxicology and Exposure, Office of Research and Development, US Environmental Protection Agency, Durham, NC, United States

**Keywords:** amphibian, conservation, risk assessment, stressor, physiology

## Abstract

Controlled laboratory experiments are often performed on amphibians to establish causality between stressor presence and an adverse outcome. However, in the field, identification of lab-generated biomarkers from single stressors and the interactions of multiple impacts are difficult to discern in an ecological context. The ubiquity of some pesticides and anthropogenic contaminants results in potentially cryptic sublethal effects or synergistic effects among multiple stressors. Although biochemical pathways regulating physiological responses to toxic stressors are often well-conserved among vertebrates, different exposure regimes and life stage vulnerabilities can yield variable ecological risk among species. Here we examine stress-related biomarkers, highlight endpoints commonly linked to apical effects, and discuss differences in ontogeny and ecology that could limit interpretation of biomarkers across species. Further we identify promising field-based physiological measures indicative of potential impacts to health and development of amphibians that could be useful to anuran conservation. We outline the physiological responses to common stressors in the context of altered functional pathways, presenting useful stage-specific endpoints for anuran species, and discussing multi-stressor vulnerability in the larger framework of amphibian life history and ecology. This overview identifies points of physiological, ecological, and demographic vulnerability to provide context in evaluating the multiple stressors impacting amphibian populations worldwide for strategic conservation planning.

## 1 Stressors

Multiple common sources of physiological stress contribute to the ubiquitous threats to amphibian populations worldwide, including disease, pollution, and habitat loss as well as combinations of these stressors (Stuart et al., 2004; Wake and Vredenburg, 2008; Foden et al., 2013; Grant et al., 2016; Green et al., 2020). Stressor impacts can be detected at the organismal level before long-term population decline is apparent. Habitat constraints are frequently observed as higher density resource competition inhibiting metamorphosis, recruitment, or reproductive success in some species (Harper and Semlitsch, 2007; Rittenhouse and Semlitsch, 2007). Disease transmission often presents as an immunological response prior to mass mortality (Ohmer et al., 2021). Pollution, likewise, can result in reduced reproductive success or growth in addition to mortality, and the chronic effects of these stressors can often be detected as systemic responses within the organism that precede impacts apparent at the population level (Thambirajah et al., 2019; Trudeau et al., 2020). Oxidative stress, compromised immunity, endocrine disruption, and altered metabolic activity are some physiological indications of perturbations in biological function that can lead to phenotypical impacts on individual fitness, with implications for population dynamics.

### 1.1 Habitat degradation

Habitat conversion, degradation, and fragmentation are the primary global causes of terrestrial biodiversity loss (Haddad et al., 2015; Newbold et al., 2015). Though global amphibian declines are linked to multiple stressors and their interactions, habitat loss typically plays an outsized role due to impacts on survival, gene flow, and dispersal (Sodhi et al., 2008). Spatial range, dispersal rates, or seasonal constraints influence population connectivity, and the abiotic conditions limiting habitat availability are projected to be less favorable in response to climate change (Sodhi et al., 2008; Funk et al., 2021). Warmer and drier conditions produced from changing climatic trends provide a direct thermal stressor and are expected to accelerate habitat loss of ephemeral wetlands (Blaustein et al., 2010; Lertzman-Lepofsky et al., 2020). Thermal stressors can geographically constrain or shift suitable aquatic (Duarte et al., 2012) and terrestrial (Hoffmann et al., 2021) ranges, particularly for cold-adapted species and microclimate-dependent life history stages with limited acclimation capacity (Frishkoff et al., 2015).

Sources of anthropogenic modifications linked to amphibian habitat loss are driven by deforestation and urbanization (Cordier et al., 2021). Continued fragmentation of amphibian populations based on their hydroregime dependency has demonstrated that periods of drought effectively isolate numerous endangered species (Zamberletti et al., 2018; Allen et al., 2020). Further, conservation of breeding wetlands is insufficient to overcome the challenges presented by anthropogenically or climatically modified habitats (Allen et al., 2020), particularly the anticipated reduction in temporary wetland inundation (Brice et al., 2022). Additionally, wetland protection depends on legal decisions that are subject to amendment or revision. Even with ample habitat available, environmental stochasticity increases variance in juvenile recruitment for species dependent on ephemeral wetlands (Greenberg et al., 2017), particularly for species with high dispersal rates and/or an energetically costly metamorphosis (Funk et al., 2021; Brooks and Kindsvater, 2022). Hydroperiod duration could have a greater impact on metapopulation persistence than pathogen or contaminant exposure (Smalling et al., 2019), specifically anomalous deluge events or multiple years of drought (Walls et al., 2013; Awkerman and Greenberg, 2022; cf. Moss et al., 2021).

### 1.2 Pathogens

In addition to the limitations of habitat availability, amphibian populations are also regulated by disease and predation. Many species require fish-free breeding ponds for sufficient reproductive success and are vulnerable to predation by aquatic insects (Ohba, 2011). Anuran species and life stages vary in inherent susceptibility and ecological likelihood of exposure to waterborne pathogens such as ranaviruses and *Batrachochytrium dendrobatidis* (*Bd*) (Haislip et al., 2011; Hoverman et al., 2011). *Bd*, the fungus responsible for chytridiomycosis, is found in cooler, lentic waterbodies (Spitzen-van der Sluijs et al., 2017), and prevalence is often highest among amphibian larvae, with later life stages more resistant to infection (Li et al., 2021). Amphibian response to chytridiomycosis often involves the complement system, in an immunological response to the pathogen, and is frequently detected through bacteria-killing assays (BKA; Rodriguez and Voyles, 2020). Ranavirus is often detected in amphibian communities with greater species diversity (Bienentreu et al., 2022). Pathogen effects can be exacerbated by transmission via more resilient invasive species that spread disease in addition to competing for the diminishing habitat of native species. For example, the American bullfrog (*Lithobates catesbeianus*) is a particularly invasive species that is less susceptible to ranavirus and chytridiomycosis-induced lethality, and therefore acts as an influential vector facilitating world-wide transmission of ranavirus (Hossack et al., 2023). The global trade and subsequent farming of this species for human consumption have resulted in the detection of ranavirus in native populations from previously uncontaminated regions such as those of Brazil and Mexico (Ruggeri et al., 2019 and Saucedo et al., 2019, respectively). International trade of the invasive *Xenopus laevis* has also contributed to the spread of chytridiomycosis (Fisher and Garner, 2020).

Amphibian species differ in their response to the fungal pathogen *Bd* with some species showing downregulation of cellular and metabolic functions and upregulation of adaptive immune gene response; however, such responses are ultimately insufficient to prevent high microbial infection loads (e.g., Eskew et al., 2018). Other species with more diverse dermal antimicrobial peptide communities showed minimal response to infection (Eskew et al., 2018). Lower temperatures may increase inflammation-related responses as opposed to warmer temperatures increasing adaptive immune responses (Ellison et al., 2020). More bacterial reads, presumably from frog microbiomes, were found in populations with a history of ranavirus (Campbell et al., 2018). Differential impacts of changing climate on host and pathogen further complicate strategies to prevent transmission (Blaustein et al., 2012). It is likely that warming climates will impact viral loads, as observed in juveniles at warmer temperatures with less intense but persistent infections (Brunner et al., 2019), and bacteria-killing ability is reduced at higher temperatures in some species (Rodriguez and Voyles, 2020). Coinfection of ranavirus and chytrid in several endemic tadpoles underscores the importance of understanding the etiology and interactions of these pathogens for effective conservation of amphibians and other aquatic vertebrates (Warne et al., 2016).

### 1.3 Pesticides

Agricultural and residential pesticide use has also been implicated as a contributing factor in declining amphibian populations (Hayes et al., 2010; Brühl et al., 2011, 2013) with agriculture identified as the most common cause of extinction threats for amphibians and other terrestrial vertebrates (Munstermann et al., 2022). A meta-analysis of pesticide effects revealed moderate impacts on survival and decreased mass and relatively greater impacts from deformities not associated with phylogeny. Although contaminants of emerging concern were underrepresented in pollutant studies, pesticide effects were comparable with those of wastewater, less impactful compared to deicer effects, and relatively greater than those of metals and phosphorus compounds (Egea-Serrano et al., 2012). Additionally, transgenerational impacts, lethal and sublethal, have been demonstrated from exposure to environmentally relevant pesticide concentrations (Karlsson et al., 2021; Usal et al., 2021). The amphibian life cycle allows complex exposure dynamics in both aquatic and terrestrial environments, and recommended application rates of many pesticides result in high mortality from terrestrial exposure (Brühl et al., 2013), although terrestrial effects are less frequently documented. Indirect effects of pesticide use at lower concentrations than those toxic to amphibians potentially impact the full lifecycle of amphibians through reduction of resources, although aquatic food web effects are more frequently reported than terrestrial food web effects (Relyea and Diecks, 2008; Relyea, 2009). Overall, aquatic pesticide exposure can alter various endocrine functions important to development and reproduction and result in a variety of systemic impacts in amphibians (Thambirajah et al., 2019). A recent review of endocrine disruption by agrochemicals summarized changes in lipid and energy metabolism among fungicides; effects on metabolism, metamorphic success, and gonadal development for some herbicides; and reduced metamorphosis from fertilizer and other pesticide exposures (Trudeau et al., 2020). Evaluating the non-lethal effects of pesticides is complicated by timing of exposure and sample collection as well as tissue type, such that measured effects vary depending on species, mechanism of action, route of exposure, and the concentration of the compound (Rohr and McCoy, 2010; Glinski et al., 2021; Seim et al., 2022). Even with the abundance of scientific support correlating pesticide exposure to declining amphibian populations, it is unrealistic that impacts of pesticide exposure will be reversed, given the moderate generation times of most amphibians, the complexity of potential exposure based on their life cycle, and the substantial proportion of croplands in protected areas associated with continuing tradeoffs between food security and conservation (Vijay and Armsworth, 2021).

### 1.4 Stressor interactions

Uncertainty surrounding individual response, species vulnerability, and exposure regime complicates risk assessment determinations of multiple stressor impacts at the landscape level (Relyea and Hoverman, 2006). For instance, co-stressors such as heat, pesticides, and parasites impact amphibian immune responses and can have synergistic effects on fecundity and post-recruitment survival (Kiesecker, 2002, Thompson et al., 2022). When anthropogenic stressors and abiotic factors synergize, the immune system is challenged (Kiesecker, 2011), and early stress experienced during development can affect resilience in later life stages (Kohli et al., 2019; Le Sage et al., 2022). Disease susceptibility can increase following herbicide exposure (Rohr et al., 2013), and lower microbiota diversity, a common result of pesticide exposure, is associated with reduced parasite resistance (Knutie et al., 2017). Anticipating potential long-term effects in response to various stressors and their interactions, which can promulgate into subsequent life stages, challenges both establishing *in situ* causality from single stressors needed for tighter regulations and effective conservation management. Ultimately, ecological risk assessment is complicated not only by a deficit of toxicological data, but also a lack of ecological data to document changes in land use, species abundance and distribution, and disease transmission that are necessary for adaptive management approaches (Womack et al., 2022). Extrinsic stressors are presented in Table 1 along with physiological measurements of these effects, endogenous processes affecting the same biochemical pathways, and life stages in which departures from typical functions are detectable and/or problematic (Figure 2).

## 2 Lifestage-specific physiology

**FIGURE 1:**
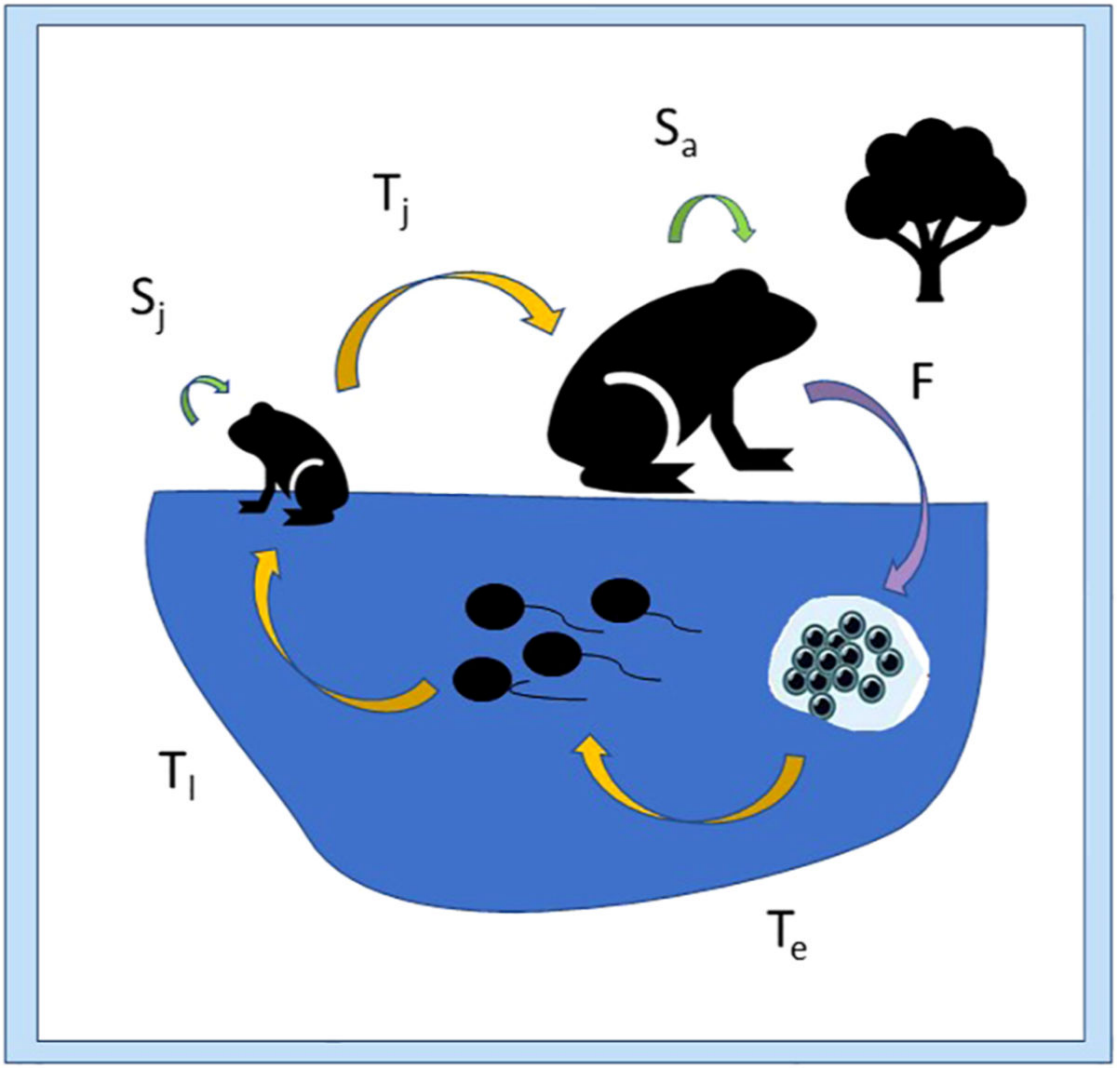
Vital rates (indicated by arrows) for the anuran life cycle, including survival rates (green arrows; S) for terrestrial juvenile (Sj) and adult stages (Sa), transition rates (yellow arrows; T) from embryo to larval stage (Te), from larval to juvenile stage (Tl), and from juvenile to adult stage (Tj); and fecundity (purple arrow; F).

**FIGURE 2:**
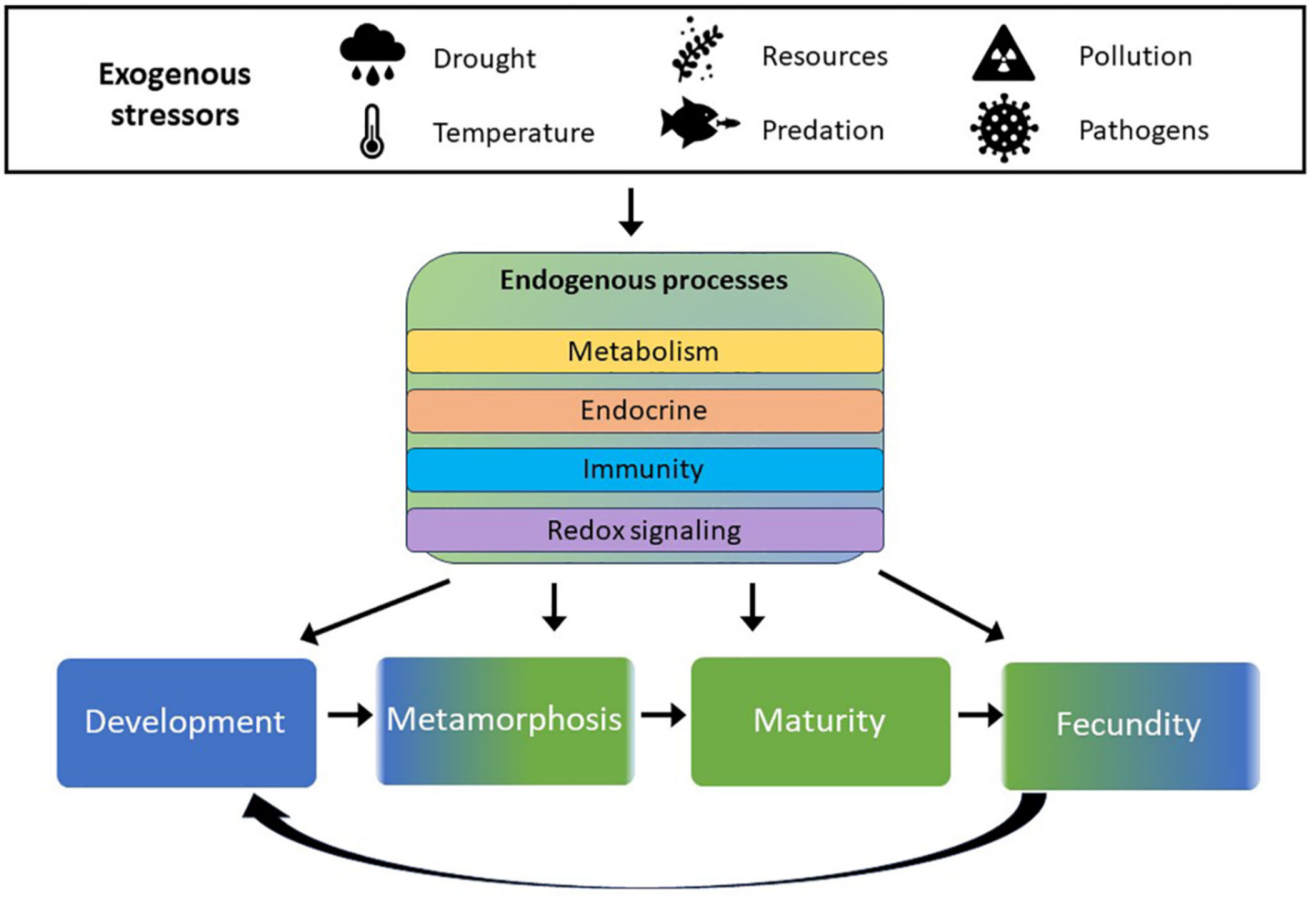
Symbols represent influential abiotic factors and extrinsic sources of population regulation. These potential stressors impact endogenous processes (shown in center block) in similar ways physiologically, and can affect individuals differently, depending on the life stage at which the stress occurs.

Effective adaptive conservation management strategies target vulnerable life stages and critical threats to wildlife populations. The biphasic life cycle of anuran amphibians makes them particularly vulnerable to extrinsic stressors because of their dependence on variable aquatic habitat resources as well as terrestrial environmental quality (Nolan et al., 2023). Their complex life history strategy and multiple potential drivers of population decline require a more nuanced approach to targeting spatial and temporal variability in stressors relative to life stage (Walls and Gabor, 2019; Awkerman et al., 2020). Distinguishing stage-specific endogenous variation in physiological processes enables anticipation of compromised physical condition in response to common stressors (Brooks and Kindsvater, 2022; Nolan et al., 2023). Here we focus on stressor impacts on transition between life stages (F, T_e_, T_l_, T_j_ in Figure 1) but present potential effects on survival and development as well.

### 2.1 Development (embryo transition to tadpole stage; T_e_)

Survival during the relatively brief stage of embryo development is largely dependent on a suitable environment to avoid predators, pathogens, or pollution, and the costs associated with such defenses differ among amphibian species and developmental mode (Brooks and Kindsvater, 2022). Amphibian clutches can experience high mortality from pathogens or predation, depending on the geographic location and ecological community composition (Kiesecker, 2011), such that habitat characteristics and regional observations are most informative in identifying these threats (Wake and Vredenburg, 2008). Pesticides aggregated as runoff in wetlands provide another potential stressor for developing embryos (Smalling et al., 2015) with lethal or sublethal impacts on individuals. Ultimately, a systematic review revealed that the time to hatching for embryos was influenced more by taxonomy and exposure to pollution, rather than experimental setting (lab vs. field; Egea-Serrano et al., 2012). Singly or in combinations, stressors during embryogenesis can lead to delayed, wide-ranging effects, resulting in a diverse array of phenotypic outcomes associated with aspects of developmental plasticity that are not observed until later life history stages (Jonsson et al., 2022).

Given the relatively brief duration of this stage in most anuran species, and rapidly changing metabolism, identifying potential stressors based on organismal condition or response could be a challenging diagnostic approach, compared to assessment of anomalous response during later life stages. Endogenous variation in embryonic metabolite levels is suggestive of energy production primarily, presumably for DNA synthesis (Vastag et al., 2011). Contaminant levels in egg masses that are linked to deformities and reduced offspring viability can result from maternal transfer of contaminants rather than indicating direct environmental exposure alone (Todd et al., 2011; Metts et al., 2013). Determining physiological response to a variety of stressors (e.g., contaminant mixtures and abiotic factors), is a complex challenge that might be approached by evaluating exposure-based epigenetic changes (e.g., DNA methylation, histone acetylation) in developing embryos (Fogliano et al., 2023) or simply assessing differential responses in later life stages.

### 2.2 Metamorphosis (transition from larval to juvenile stage; T_l_)

The morphological restructuring for transition from larval stage to juvenile stage is dependent on endocrine drivers, specifically surges in thyroid hormones (TH), regulated by thyroid hormone receptors and retinoic acid receptors (TR and RXR, respectively; reviewed in Paul et al., 2022). Endocrine regulation and body morphogenesis during the larval stage are controlled by the hypothalamic-pituitary-thyroid (HPT) and hypothalamic-pituitary-adrenal/interrenal axes (HPA/HPI) as well as the hypothalamus-pituitary-gonadal (HPG) axis (Duarte-Guterman et al., 2014). Development and metamorphosis are regulated largely by the release of the thyroid hormones thyroxine (T4) and tri-iodothyronine (T3) and modulated by the corticosteroids (CS) corticosterone (CORT) and aldosterone (ALDO; Denver, 2009). Regulation of TH signal involves cellular processes of deiodination, glucuronidation, and sulfation (Thambirajah et al., 2019). Metabolism and cardiac functions associated with development and metamorphosis are also regulated by CS. Ruthsatz et al. (2020) showed that certain metamorphic stages were significantly more susceptible to changes in growth and development due to increased TH levels, with high TH levels associated with reduced weight and size in tadpole and froglet stages as well as increased heart rate and reduced energy stores across all stages.

TH inhibition or impairment can delay development, while CS production is often associated with accelerated metamorphosis in response to pond drying or other stressors (Sachs and Buchholz, 2019; Thambirajah et al., 2019), although the role of CORT as a homeostatic response to stress is complex. The corticotropic releasing hormone (CRH) regulates the HPA axis as well as the HPT axis, thereby contributing to additional crosstalk between these pathways and circulating hormone levels (Thambirajah et al., 2022). CORT levels in southern leopard frogs increased with exposure to multiple aquatic stressors, specifically a nitrogenous fertilizer, a pesticide, and salt (Adelizzi et al., 2019). However, relatively elevated CORT levels were associated with populations less tolerant to contaminant exposure, such that differences in stress response could be indicative of exposure history (Shidemantle et al., 2022). Predator detection can also elicit an increased CORT response (Narayan et al., 2013). Signals of agrochemical disruption of endocrine function among interactions of the thyroid, gonadal, and metabolic axes in amphibians was reviewed in detail by Trudeau et al. (2020). Early life stage stressors that elevate CS production can alter endocrine response throughout the lifecycle of the individual (Denver, 2009).

Stressor perturbations in endocrine functions are particularly impactful in metamorphosing amphibians and can influence immunity, survival, and fecundity in subsequent terrestrial life stages (Kiesecker, 2002; Kohli et al., 2019). A meta-analysis determining effects on time to metamorphosis found taxonomy, pollution, and timing of exposure to be more influential than the experimental setting (Egea-Serrano et al., 2012). Pond drying constraints influencing larval development are expected to be impacted in various ways by climate change, depending on regional location (Walls et al., 2013). The duration of larval stage and developmental mode, combined with community dynamics between larval competitors and predators, can distinguish species resilience and response to such unpredictable environmental stressors (Belden et al., 2010; Moss et al., 2021; Brooks and Kindsvater, 2022). Interannual variability in hydrologic regime at temporary wetlands determines the length of the developmental period and the density of developing anuran larvae (e.g., Pechmann et al., 1989). Developmental plasticity in metamorphic climax allows variable phenotypic response to interannual conditions and is driven by the neuroendocrine processes responsible for the development of the immune system, highlighting a potential tradeoff between accelerated development and resistance to disease and parasites (Kohli et al., 2019). Likewise, tradeoffs between development and microbiota diversity or immunology are demonstrated later in life with increased susceptibility to pathogens (Le Sage et al., 2022). As northern leopard frog tadpoles approach metamorphic climax, tail tissue decreases expression of mitochondrial energy genes and upregulates expression of immunity genes (Row et al., 2016). Post-metamorphic immune function may be compromised in amphibians experiencing shorter hydrologic regimes (Brannelly et al., 2019), which may further exacerbate disease susceptibility. Therefore, interannual variance in aquatic habitat suitability can have lasting impacts on cohort fitness.

During metamorphic climax, metabolic activity changes, reflecting increasing energy needs, anabolic requirements, and tail apoptosis; these energetic requirements and fasting effects create a vulnerable transition from larva to juvenile in which contaminant body burdens can amplify (Rowland et al., 2023). The aquatic phase of the amphibian life cycle is also susceptible to reduced growth in response to pathogens that have been introduced to waterbodies, although survival is rarely impacted at this stage (Nolan et al., 2023). Although phylogeny and abiotic environmental variables determine the initial likelihood of *Bd* or ranavirus occurrence in areas of viral compatibility, other stressors such as pesticides can further influence viral loads and the resistance of the host population as well as the prevalence of parasite-induced deformities (e.g., Kerby et al., 2011; Kiesecker, 2011; Pochini and Hoverman, 2017).

### 2.3 Maturity (juvenile transition to reproductive adult; T_j_)

The literature on this critical amphibian life stage is scarce, due in part to the complexity of rearing and maintaining juvenile amphibian populations in a laboratory setting through maturation, as well as the challenge of monitoring individual juvenile amphibians from metamorphosis through reproduction in a field setting (see Petrovan and Schmidt, 2019). Furthermore, a systematic analysis of factors affecting survival found pollutants and the experimental setting (lab vs. field) to be more influential than taxonomic group or developmental stage in the study (Egea-Serrano et al., 2012). Although pre-metamorphic environmental conditions directly influence post-metamorphic life stages, there may also be distinctly different age or stage-specific stress responses in amphibians, as evidenced by variations in sucrose and starch pathway regulation following pesticide exposure in larval and juvenile amphibians (Zaya et al., 2011; Dornelles and Oliveria, 2016; Van Meter et al., 2018). Survival to reproduction was positively correlated with lipid stores at metamorphosis among two *Ambystoma* salamander species, and lipid stores were greater among individuals emerging from longer hydroperiod wetlands. Furthermore, total rainfall during years of juvenile development was also positively associated with survival to reproduction (Scott et al., 2007).

Potential carry-over effects from compromised development can exist (Kiesecker, 2002; Rohr et al., 2006; Kohli et al., 2019) with additional risk from stressors in the terrestrial environment. Terrestrial habitat degradation and habitat fragmentation influences the connectivity of both amphibian populations and their potential pathogens (Stevens and Baguette, 2008; Costa et al., 2021; Becker et al., 2023). Reduced skin and gut microbiota in the larval stage can also reduce parasite resistance in adults (Knutie et al., 2017). Amphibian skin contains antimicrobial peptides linked to immunity and defense functions as well as to biosynthesis and metabolism (Huang et al., 2016). Skin secretions have demonstrated antimicrobial antioxidant properties and can be beneficial to healing (Wang et al., 2020). Bacterial and fungal taxonomy in skin secretions is associated with disease resistance (Bates et al., 2022), with the skin microbiome affected by the same abiotic factors that influence *Bd* occurrence (Ruthsatz et al., 2020). Some species’ secretions contain sufficient toxins to be lethal to predators, thereby reducing mortality via predation (Liu et al., 2022). Compromised skin microbiome diversity is implicated in important physiological functions such as electrolyte and hydration loss, disease susceptibility, and increased pesticide effects. Pesticide exposure has been linked to disruption of the skin microbiome and antimicrobial peptides of amphibians, thereby affecting vulnerability to pathogens (Krynak et al., 2017; McCoy and Peralta, 2018; Jiménez et al., 2021). Habitat disturbance has also been associated with skin microbiome diversity, primarily via pathogen dispersal (Neely et al., 2022), underscoring the ecological complexity of proximate mechanisms of multiple stressors and their potential interactions. Enhanced data collection efforts on juvenile amphibians are essential to improve risk assessment and inform management decisions at the local scale.

### 2.4 Fecundity (adult production of embryos; F)

Reproductive failure associated with insufficient hydroperiod is a determinant of lifetime reproductive success in species dependent on ephemeral wetlands for breeding (Taylor et al., 2006; Stevens and Baguette, 2008). In years with suitable hydroregime, terrestrial density dependence and sex ratio can affect fecundity within a population (Kissel et al., 2020; Baranek et al., 2022). Effects of endocrine disruption in developmental phases as well as during gamete production could also reduce fecundity via altered gonadal development, or a high incidence of intersex individuals in the population (Lambert et al., 2015; Marlatt et al., 2022). Sex-specific age at maturation could further restrict operational sex ratio in amphibian populations (Baranek et al., 2022). In addition to the endocrine disruption associated with xenobiotic exposure, temperature can affect sex determination, with potential impact on operational sex ratio following extended periods of anomalous temperatures (Lambert et al., 2015; Ruiz-García et al., 2021). The lasting impact of such shifts will vary depending on the species life history and genetic sex determination (Bókony et al., 2017).

## 3 Field-based measures of stressor response

Assessing the status of a wildlife population or relative condition of an individual within its habitat is a challenge complicated by the heterogeneity of both organismal response and stressor distribution. Acquiring an adequate sample size for a meaningful detection of environmental or stressor effects could limit the practical scope of most field-based efforts, while standardization of conditions can bias the interpretation of stressor response in most laboratory or mesocosm settings. Stage-specific physiology, along with ecological or life history vulnerabilities, provides additional context for interpretation of potential stressor effects (Venturino et al., 2003). For example, intestinal development and tail resorption in larvae are coincident with signs of oxidative stress (Menon and Rozman, 2007). Establishing baseline physiology with common biomarkers provides context of endogenous variability during the amphibian life cycle. Identifying these informative endpoints and sensitive stages can preclude the need for extensive accounting of stressor-specific effects and interactions.

### 3.1 Physiological processes

Systematic responses to stress include endocrine disruption, oxidative stress, metabolic perturbation, and compromised immunity (Venturino et al., 2003). Specifically, elevated CORT and standard metabolic rate as well as decreased antioxidant enzymes are common signals of abiotic and xenobiotic physiological stress (Burraco and Gomez-Mestre, 2016). Endocrine disruption in the interconnected hormonal axes can also trigger responses in other systems, such as neurodegenerative effects, oxidative damage, impairment of mitochondrial function, and teratological effects (Di Lorenzo et al., 2020). Taxonomic family and pollutant exposure were significant determinants of developmental abnormalities in a systematic review of ecotoxicological studies (Egea-Serrano et al., 2012), and specific phenotypical abnormalities can be ascribed to different classes of chemicals (Venturino et al., 2003).

Endocrine-driven developmental processes are highly conserved in vertebrates (Paul et al., 2022), as are many physiological endpoints associated with both homeostatic and lethal responses to pesticides and contaminant exposure. Larval amphibians are especially susceptible to endocrine disruption due to their reliance on hormonal cues for initiation and timing of metamorphosis and sex determination (Duarte-Guterman et al., 2014). Crosstalk between hormonal axes includes an evolutionary history of HPT and HPG signaling (Thambirajah et al., 2022). Genetic sex determination during developmental stages varies between and within amphibian species due to rapid turnover of genes such that either males or females can be heterozygotic, with some species having three sex chromosomes (Miura, 2017). Sex reversal in response to external temperature or steroid hormones can also affect the sex ratio of a cohort (Roco et al., 2021). Although estrogenic and androgenic effects have been studied much more extensively in mammals, intersex amphibian larvae resulting from reproductive steroid hormone exposure have been associated with effects on the androgen receptor (AR) and thyroid receptor (TR) and altered expression of dio1, dio2, dio3, and thrb (Thambirajah et al., 2022). Increased vitellogenin production is a common indication of feminization (Venturino and de D’Angelo, 2005), and increased formic acid has been suggested as an indicator of androgen receptor binding and anti-androgenic effects in larvae (Melvin et al., 2018).

Endogenous changes in metabolism are also associated with lifecycle-dependent physiological processes (Ichu et al., 2014). During metamorphosis, metabolic pathways are dramatically altered in the liver and the tail as a result of lipid and carbohydrate metabolism (Zhu et al., 2020). Metabolomic changes during metamorphosis demonstrate physiological processes associated with morphological restructuring in metabolic pathways, including the urea cycle as well as arginine and purine/pyrimidine, cysteine/methionine, sphingolipid, and eicosanoid metabolism; however, similar metabolite expression in humans is associated with disease (Ichu et al., 2014). As the tadpole tail regresses and intestines restructure, lipid peroxidation is increased; depleted catalase (CAT) and glutathione contribute to oxidative stress, as demonstrated by CAT, superoxide dismutase (SOD), and malondialdehyde (MDA) expression; and the antioxidant ascorbic acid increases as organs develop (Menon and Rozman, 2007; Guo et al., 2022). Epidermal galactose levels, and specifically 25 uniquely expressed genes, are predictive of chytrid outbreaks and are life stage dependent, with higher expression at metamorphosis (Wang et al., 2021). Food constraints in the juvenile stage were associated with higher lipid peroxidase and lower SOD, glutathione S-transferase (GST), glutathione peroxidase, glutathione and sulfhydryl groups (Prokić et al., 2021).

Antioxidant system response (AOS) and oxidative stress is highest at metamorphic peak, and associated with lower glutathione, CAT, glutathione peroxidase, GST, and sulfhydryl groups, and oxidative stress is exacerbated by decreasing water levels (Petrović et al., 2021). Hepatic GST activity has been proposed as a biomarker indicative of TH signaling imbalance and developmental effects (Chen et al., 2017). Upregulated pathways include transamination and the urea cycle because of hepatic catabolism, TCA cycle and oxidative phosphorylation resulting from energy metabolism (although these are downregulated in the tail), and hepatic glycogen phosphorylation and gluconeogenesis (Zhu et al., 2020). Decreased activity occurred in β-oxidation and the pentose phosphate pathway, and downregulation of glycolysis, β-oxidation, and transamination in the tail accompanied reduced protein synthesis and lower energy production and consumption, although glycogenesis, fatty acid elongation and desaturation, and lipid synthesis were maintained (Zhu et al., 2020).

Indication of oxidative stress is a common detoxification response to many chemical classes and is characterized by altered expression of mixed function oxidases (MFO; e.g., CYP1A, EROD, demethylase), GSH, lipid peroxides, and antioxidant enzymes (CAT, SOD; Venturino and de D’Angelo, 2005). Oxidative stress and lipid peroxidation, as demonstrated by increased SOD and CAT activity were also associated with hepatotoxicity resulting from increasing organophosphate exposure, although GST activity was unchanged, and MDA decreased (Li et al., 2017). Common indications of oxidative stress as a detoxification response include glutathione deficits and production of GST (Venturino and de D’Angelo, 2005). Interactive oxidative stress effects of pesticide concentration and parasite abundance were observed in thiol levels of recent metamorphs, with nematode infection related to elevated thiol and catalase expression (Marcogliese et al., 2021).

Amphibian physiological responses to environmental stressors have been well documented (Park and Do, 2023), and the various threats that impact amphibian populations can elicit similar physical effects. For example, xenobiotic exposure or threatening environmental conditions (e.g., pond drying or predator presence) is commonly associated with oxidative stress and production of reactive oxygen species (ROS; Burraco et al., 2017). Habitat fragmentation and degradation, coincident with anthropogenic infringement and climate change, contribute to invasive species introduction, disease outbreak, and increased pollution, multiplying threats to immunocompetency (Kiesecker, 2011). Immune functions impacted by common amphibian stressors and their interactions are indicated in various matrices and measurements. For example, glucocorticoids (GC) are CS hormones influencing the immune system, tissue inflammation, and cardiovascular response (Rollins-Smith, 2017), and frequently indicate physiological stress. However, some stressors, e.g., food deprivation, can yield differential endocrine responses, with reduced CORT in juveniles conserving energy resources as opposed to increased CORT levels in food-deprived tadpoles (Crespi and Denver, 2005).

### 3.2 Omics technologies

Stressor-specific measurements of organismal response introduce complexity to both laboratory and field-based assessment approaches, as well as to the interpretation of multi-stressor scenarios. Evaluating biomarker expression can help identify biochemical perturbations indicative of systemic stress to environmental conditions. The suite of ‘omics technologies, including genomics, transcriptomics, proteomics, and metabolomics, can shed light on the underlying biological processes and provide a means to identify specific genes, metabolites, and pathways that are affected in an amphibian ecological risk assessment.

Comparative genomics is increasingly recognized as a valuable tool for conservation purposes (Kosch et al., 2023). This includes the use of reference genomes in eDNA approaches for monitoring populations (Breton et al., 2022; Saeed et al., 2022), informing genetic rescue efforts for threatened amphibians (Kosch et al., 2023), and using sequence information to predict protein similarity and infer ecotoxicological implications across species (LaLone et al., 2023). However, compared to other vertebrate classes, genomic coverage for amphibians is currently recognized as lacking (Hotaling et al., 2021). Kosch et al. (2023) provided a review of the 32 available amphibian reference genomes and found variable annotation quality for the available genomes and uneven coverage across amphibian families, with genomic comparison further complicated by the presence of large, repetitive genomes. This limited availability of amphibian reference genomes presents challenges for generalizing ecotoxicological results to higher taxonomic levels within the class Amphibia. Despite these challenges, genetic approaches can provide conservation insights. This is particularly true for amphibian species with cryptic habits and biphasic life cycles, which complicate traditional field-based measurements of survival, fecundity, and migration (Mazerolle et al., 2007; Funk et al., 2021). Landscape genetics, for instance, can reveal connectivity within a population as well as isolated subpopulations (Watts et al., 2015). This information can then be used to prioritize conservation targets for threatened amphibians (e.g., Forester et al., 2022). The pressing need for increasing knowledge of amphibian genomes to assist in conservation efforts was highlighted by Calboli et al. (2011). It is hypothesized that only a relative few, simple mechanisms of gene alterations are indicated in amphibians’ response to numerous environmental stressors. Functional genomics has been used to probe the molecular underpinnings of field observations concerning the sexual differentiation in amphibians (Bögi et al., 2002), fragmentation of populations (McCartney-Melstad et al., 2018), and pathogen-host interactions (Zamudio et al., 2020).

In the laboratory, transcriptomics approaches leverage differential gene expression (DGE) approaches by contrasting the expression level of transcripts in stressed individuals versus control individuals. Changes in gene expression can reveal which genes are upregulated or downregulated, thereby identifying perturbations in specific biochemical pathways regardless of the origin of the stressor. The magnitude of the response could indicate functional points of departure (e.g., Ewald et al., 2022; Mittal et al., 2022). Transcriptomics data, generated from controlled laboratory exposures, provide a comprehensive view of gene expression changes comparable to traditional apical endpoints. The large volume of data, coupled with the fact that the expression responses are specifically associated with the mechanism of the stressor, suggests the possibility of developing expression-based “fingerprints” or signatures resulting from single and multi-stressor exposures. These can be used to determine if the magnitude of an exposure to a toxicant or stressor of a particular mode of action is likely to elicit biological perturbations that can be linked to or predictive of apical effects. Furthermore, high-throughput transcriptomics (HTTr) methods have been developed to observe changes in gene expression in cells, rather than in test species, after exposure to chemicals (Schirmer et al., 2010). These methods are less resource-intensive than traditional toxicity testing and can be used to determine at what concentration chemicals impact cellular biology and to develop adverse outcome pathways (AOPs). For vertebrates, such regulatory testing programs aim to evaluate the potential endocrine-disrupting effects of chemicals, utilizing the conservation of certain endocrine pathways among vertebrate classes to evaluate the feasibility of extrapolating data across taxa. These approaches integrate functional genomics with transcriptomics to establish the confidence levels in pathway conservation while identifying the specific needs for additional data to advance read-across methods for estrogen, androgen, thyroid, and steroidogenesis pathways in vertebrate ecological receptors (McArdle et al., 2020).

Metabolomics technology may also provide a means to address the uncertainties surrounding chemical risk assessment of single and multiple stressors. Available technology measures the changes in hundreds (if not thousands) of metabolites simultaneously, effectively capturing a metabolomic fingerprint as a snapshot of an organism’s altered physiology. This metabolomic fingerprint of subcellular biological responses often represents immediate or early response within the organism to stresses and can be associated with signaling networks that are linked to adverse outcomes at higher levels of biological organization. Successful application of metabolomics to differentiate multi-stressor response was achieved by Snyder et al. (2022). Similarly, the use of transcriptomics and proteomics for advancing amphibian toxicogenomic studies was reviewed in Helbing (2012). Relying on ‘omics technologies to identify meaningful suites of stressor response and target demographic vulnerabilities for sample collection could offer a comparative physiology approach for detecting impacted individuals and populations.

Exogenous impacts of xenobiotic exposure can exacerbate stressors that accompany particular life stages. Reduction in body size during metamorphosis and fasting during hibernation result in increased metabolic demands and greater body burdens of contaminants due to biomagnification (Rowland et al., 2023). Aquatic exposures of various pesticides were associated with increased galactose metabolism and lactose degradation, indicating effects on energy metabolism (Glinski et al., 2021). Pathways associated with glucogenesis and glycolysis were also indicators of energy metabolism impacts in terrestrial juvenile frog exposures (Van Meter et al., 2022). The urea cycle was frequently impacted by various pesticides, and the purine metabolism pathway was also enriched, indicating increased energy production as a response to toxicity. Reduction in glutathione levels is another common result of pesticide exposure indicative of oxidative stress in both larval and juvenile amphibians (Ichu et al., 2014). Reduced citrate, α-ketaglutarate, and fumarate were also proposed as oxidative stress biomarkers, as intermediates of the tricarboxylic acid cycle (Melvin et al., 2018).

The magnitude of altered metabolite regulation during later life stage terrestrial exposures was not indicative of bioaccumulation, and exposure to combinations of pesticide did not always have a synergistic effect on juvenile toads (Van Meter et al., 2018). In fact, extrinsic sources of urea as fertilizer at low concentrations can counteract combined pesticide effects, presumably by facilitating excretion and detoxification, although excessive doses can be detrimental (Van Meter et al., 2022). Hepatic metabolome analyses revealed altered pathways indicating stress caused by both predation and pesticide exposure; these include aminoacyl-tRNA biosynthesis, galactose metabolism, glutathione metabolism, and arginine biosynthesis (Snyder et al., 2022). Transgenerational fertility effects of endocrine disruption due to pesticide exposure were associated with greater mass, increased palmitoleic:palmitic acid ratio, and decreased glucose (Karlsson et al., 2021). As studies of multistressor deviations from normal metabolite activity continue, identification of meaningful pathway perturbations could provide a systematic method of identifying locations of environmental impacts without prerequisite knowledge of specific land use changes or fate and exposure of particular pollutants.

### 3.3 Sampling strategies

Traditional measures of contaminant impacts on amphibians focus on body burden concentrations and somatic indices of body condition (e.g., Băncilă et al., 2010) as well as general indicators of genotoxic and mutagenic impacts (i.e., comet and micronucleus assays) and more targeted analyses of cellular-level endpoints. For larger taxa (e.g., birds and mammals), marking individuals and collecting tissue samples can be a routine, noninvasive procedure conducted in the field to inform physiological condition. However, the small body size of amphibians hinders repeated sampling of blood or plasma for various analyses. For smaller amphibians, sampling techniques might involve toe-clipping for both individual identification as well as tissue collection, or sample collection might require sacrifice of individuals, particularly at earlier life stages.

Many common assays measure hematological enzymatic responses typical of exposure to specific xenobiotic contaminants (Ohmer et al., 2021). For example, esterase activity (acetylcholinesterase, butyryl cholinesterase, and carboxyl esterase) can indicate potential developmental effects, but response varies greatly within and between species (Venturino et al., 2003; Venturino and de D’Angelo, 2005). Hematological measures representative of immune response include leukocytes, neutrophil/lymphocyte ratio, bacterial killing assays, and delayed hypersensitivity assays (Ohmer et al., 2021). Changes in neutrophils and lymphocytes are often proportional with increased glucocorticoid levels, indicating physiological stress; however, neonicotinoid exposure altered leukocyte profiles relative to neutrophils or eosinophils but did not affect CORT levels in northern leopard frogs (Gavel et al., 2021). Neutrophil to lymphocyte ratios were also a good indication of environmental stressors and were associated with total dissolved solid levels in aquatic habitats that impacted growth and development (Ruso et al., 2021). Combinations of physiological indices are also informative to link endpoints with individual condition (e.g., Park et al., 2021).

Several minimally or non-invasive techniques that may be more effective for amphibians are now routinely used including urinalysis, fecal sampling, waterborne sampling, salivary swabs, and dermal swabs (Narayan et al., 2019). Among these techniques, saliva has only been validated in adults and not juveniles. Janin et al. (2012) concluded that CORT concentrations in saliva were highly correlated with urine measurements in toads. Urinalysis has been previously used to track endocrine response such as the reproductive hormones estradiol, progesterone, and testosterone within both captive and wild caught amphibians (Narayan, 2013). Additionally, CORT levels can be quantified from urine to identify differences in stress response due to predation risk or pathogen prevalence (Kindermann et al., 2012; Narayan et al., 2019). While urine samples have the distinct advantage of being highly concentrated for measuring endocrine functions along with physiological stress, the method is not always ideal for smaller amphibians producing lower volumes (Narayan, 2013; Baugh et al., 2018).

Another minimally invasive technique for endocrine analysis for amphibians of any size is immersing the individual in a clean container of water for a designated length of time, after which the released hormones can be quantified from the water. Most studies have used this technique to measure CORT release rates, which have been validated with circulating plasma levels (Gabor et al., 2013a). Waterborne sampling has evaluated CORT release rates in the presence or absence of *Bd*, predation, or pesticide exposure (Gabor et al., 2013b; Van Meter et al., 2019; Snyder et al., 2022) and can also be indicative of nitrate stress in amphibians (Ruthsatz et al., 2023). CORT is a potentially useful biomarker for amphibians to indicate stress, and non-invasive sampling methods offer the potential of serially sampling the same individual (Narayan et al., 2019; Tornabene et al., 2021). Therefore, within a short timeframe baseline values and acute elevation in CORT are quantifiable (Hammond et al., 2018). Szymanski et al. (2006) collected feces of adult anurans to quantify reproductive hormones, enabling sex identification. In addition to CORT, two other reproductive hormones, progesterone and estradiol, have also been quantified in water from amphibians (Baugh et al., 2018).

While dermal swabbing is most notable for detecting the presence of pathogens in amphibians (e.g., Standish et al., 2018), more recent studies have expanded on what can be tested in amphibian mucus, such as DNA collection and glucocorticoids (Poorten et al., 2017; Narayan et al., 2019; Santymire et al., 2021). A lab-based salamander study examined the difference between liver metabolomics and dermal swab metabolomics, to determine if similar pathways are impacted in the presence of pesticides, measuring for presence/absence of pathogens and glutathione as well (Van Meter et al., in press). Dermal swabs enable *in situ* field sampling with minimal handing time, which is particularly advantageous for threatened and endangered species and allows serial sampling of the same individual or environment (Santymire et al., 2018, 2021; Scheun et al., 2019; Tornabene et al., 2021).

Sampling techniques that are well-established at the individual level can also provide a comparison, through DGE or hepatic metabolites, of localized stressor response indicative of differential population-level effects. Such response-based metrics could preclude the necessity to anticipate synergies, compensation, and interactions between ubiquitous stressors that might be heterogeneously distributed. Rather than spatially explicit analysis of stressors within the species distribution, identifying variance in relative response within the population could target conservation concerns more rapidly within a diverse landscape while accounting for baseline fluxes in physiology. For example, the complex physiological changes during metamorphosis comprise endocrine interactions and changes in energy allocation as tail resorption and leg growth occurs, such that tissues sampled could vary in the cellular-level activity. Stage-specific fluxes in endocrine activity also affect response observed in individuals, such that standardizing measurements within consistent developmental stages is advisable when sampling larvae. Field-based observations could also be influenced by the environment of the individuals, making observations about water quality, larval density, and community composition relevant to evaluating stress response. As measures of individual response are considered within appropriate life stage time points, comparable evaluation of location-specific perturbations in baseline physiological functions can guide more targeted conservation actions.

## 4 Discussion

As amphibians are impacted by multiple stressors and their interactions, the capability to assess cumulative impacts on biochemical pathways within an organism’s native habitat facilitates quantification of exposure risk and possible additive or synergistic effects. However, even at the organismal level, amphibians often lack sufficient toxicology data for evaluation of cellular-level responses (Marlatt and Martyniuk, 2017), and variance in individual response and chronic exposure obscure definitive metrics of detrimental effects on the population. Additional research is needed to identify reliable biomarkers that are consistently indicative of points of departure from normal cellular function in response to environmental stressors. Standardized indices of biomarker perturbations in response to stressors enables identification of reliable predictors of long-term impacts (Pham and Sokolova, 2023); however, caution must be taken to verify cellular responses are linked to demographic effects, rather than a homeostatic response to stressors (Forbes et al., 2006). Evaluating potential threats linked to synergistic exposure effects (e.g., reduced dermal microbiota) or multiple exposure routes (i.e., aquatic and terrestrial) requires situation-specific ecological context. Implementation of weight of evidence effects could further classify cumulative threat levels of variable biomarker responses (Cecchetto et al., 2023). An initial step towards multi-stressor risk assessment is outlined here, namely by exploring stage-specific variance in biochemical pathways and identifying points of physiological vulnerability in the life cycle as a screening-level conservation approach.

## Figures and Tables

**TABLE 1:** Endogenous activity associated with transitional phases of the anuran lifecycle, exogenous stressors that alter biological processes, methods to assess organismal effects, and potential demographic impacts. ↑ upregulation or increased expression; ↓ downregulation or decreased expression.

Stage	Systemic response	Endogenous activity	Exogenous stressors	Assessment methods	Demographic endpoint
**Embryolarval development**	Metabolism	Energy production, DNA synthesis, protein synthesis↑, alanine ↓ aspartate ↓, α-ketoglutamine ↑ (Vastag et al., 2011)	Aquatic conditions, including chemical pollution, pathogens, predation (Kiesecker, 2011)	Whole-organism metabolites (Vastag et al., 2011)	Embryo mortality; cessation of development
**Metamorphosis**	Metabolism	Increasing energy needs, anabolic activity, tail apoptosis; greater dehydration from fasting (Rowland et al., 2023); purine, arginine and pyrimidine, urea cycle metabolites, arginine and purine/pyrimidine, cysteine/methionine, sphingolipid, and eicosanoid metabolism (Ichu et al., 2014)	↑ galactose metabolism and lactose degradation with xenobiotic exposure (Glinski et al., 2021) or reduced resources; ↓ glutathione (Ichu et al., 2014); galactose predictive of chytrid (Wang et al., 2021); predation and pesticide exposure alter aminoacyl-tRNA biosynthesis, galactose and glutathione metabolism, arginine biosynthesis (Snyder et al., 2022); pesticide exposure impacts serine and threonine, histadine, linoleic acid, and sphingolipid metabolism	Whole-organism or tissue metabolomics (Ichu et al., 2014)	Delayed development, reduced transition to juvenile stage
	Redox signalling	Lipid peroxidation ↑, glutathione ↓, catalase ↓, SOD, CAT, MDA expression altered during intestinal development and tail resorption, ascorbic acid ↑ for collagen synthesis (Menon and Rozman, 2007; Guo et al., 2022); glutathione peroxidase ↓, GST ↓, sulfhydryl groups ↓ (Petrović et al., 2021)	Lower antioxidant activity and increased lipid peroxidation to xenobiotics or environmental conditions (abiotic or density effects; Burraco et al., 2017; Petrović et al., 2021); ↑ thiol and CAT in pesticide and nematode infection (Marcogliese et al., 2021)	ROS production in tissues; antioxidant enzymatic responses of SOD, CAT, MDA, GST (Menon and Rozman, 2007; Chen et al., 2017; Guo et al., 2022); decreased expression of GSH; increased TBARS	Delayed development; reduced transition to juvenile stage
	Endocrine response	CS in response to ↑ TH, regulate development via diodination, glucuronidation, sulfation, affecting HPT, HPA, HPG axes (Denver, 2009; Duarte-Guterman et al., 2014; Thambirajah et al., 2019)	GC ↑ to some xenobiotics (Burraco and Gomez-Mestre, 2016; Trudeau et al., 2020), environmental conditions (Sachs and Buchholz, 2019; Thambirajah et al., 2019), predators (Narayan et al., 2013) ; neurogenerative, oxidative, mitochondrial, teratological effects (Di Lorenzo et al., 2020)	CS and TH levels in tissue or immersed water (Gabor et al., 2013a); tissue/organism enzyme activity or DGE in AR, TR, tra, trb, dio2, dio3 (Thambirajah et al., 2022); ambient water assay; size at metamorphosis (Rowland et al., 2023); vitellogenin indicative of feminization (Venturino and de D’Angelo, 2005)	Time to metamorphosis; cohort sex ratio; carryover to juvenile immunity, survival, fecundity (Kiesecker, 2002, Denver, 2009; Kohli et al., 2019; Ruthsatz et al., 2020; Le Sage et al., 2022)
	Immunity	Endocrine-driven development of immunity; immunosuppression at metamorphosis (Rollins-Smith, 2017)	Viral loads, resistance, and parasite prevalance affected by pesticides and abiotic factors (Kerby et al., 2011; Kiesecker, 2011; Knutie et al., 2017; Pochini and Hoverman, 2017); Microbiome in tadpoles impacted by xenobiotic exposure	Gut microbiome diversity; at advanced developmental stages – blood leukocytes, white cell lymphocytes and granulocytes (basophils, neutrophils, eosinophils); DGE (Row et al., 2016)	Reduced survival due to pathogens and parasites (Kiesecker, 2011)
**Juvenile maturation to adult**	Endocrine	TRH influences TSH (Paul et al., 2022)	Food constraints ↓ CORT (Prokić et al., 2021); variance in CORT along latitudinal cline (Le Sage et al., 2022)	Dermal swab, fecal content, tissue or ambient water assay of CS (Gabor et al., 2013a)	Behavioral responses to stressors, reduced dispersal
	Immunity	Gut microbiome linked to resistance of parasites (Knutie et al., 2017), skin microbiome linked to resistance of pathogens (Krynak et al., 2017; McCoy and Peralta, 2018; Jiménez et al., 2021); possibly compromised by shortened developmental hydroperiod (Brannelly et al., 2019); Lower juvenile immunity relative to mature adults	Dermal microbiome and pathogen vulnerability impacted by xenobiotic exposure (Krynak et al., 2017; McCoy and Peralta, 2018; Jiménez et al., 2021); habitat degradation affects vulnerability to pathogens (Stevens and Baguette, 2008; Costa et al., 2021; Becker et al., 2023)	Microbiome diversity in skin mucosa (Neely et al., 2022); antimicrobial peptides (Huang et al., 2016); white cell lymphocytes and granulocytes (basophils, neutrophils, eosinophils); B and T cells in organs, MHC-II; antibodies - IgA/X, IgD, IgF, IgM, IgY	Susceptibility to pathogens, reduced juvenile survival or limited dispersal due to disease or deformities (Kiesecker, 2002, Rohr et al., 2006; Krynak et al., 2017; Kohli et al., 2019)
	Metabolism	Related to endocrine activity; longer hydroperiod ↑ lipid stores (Scott et al., 2007)	Influenced by temperature, water loss, xenobiotics; food deprivation ↓ CORT; pesticides altered sucrose and starch pathway regulation (Zaya et al., 2011, Dornelles and Oliveria, 2016, Van Meter et al., 2018)	Body condition; energy metabolism in tissue	Reduced juvenile survival
	Redox signalling	Increased antioxidants during estivation in preparation for oxidative stress; lower oxidative metabolism enzyme activity during estivation (Rowland et al., 2023)	Food constraints ↑ lipid peroxide; ↓ SOD, glutathione peroxidase, GST, glutathione and sulfhydryl groups (Prokić et al., 2021); pesticides and pathogens ↑ thiol; nematode infections ↑ thiol, ↑ catalase (Marcogliese et al., 2021)	ROS production; antioxidant enzymatic responses of SOD, glutathione peroxidase, glutathione, GST, thiol, catalase (Marcogliese et al., 2021; Prokić et al., 2021)	Survival to following breeding season, potentially a function of size/condition at end of season
**Adult fecundity**	Endocrine activity	Gonadotropins released by pituitary; estrogen, androgen, progestogen regulate reproduction (Duarte-Guterman et al., 2014)	Endocrine disrupting compounds can disrupt gonadal development, sexual differentiation (Lambert et al., 2015; Marlatt et al., 2022); temperature impact on sex determination (Lambert et al., 2015; Ruiz-García et al., 2021); density-dependent resource availability (Kissel et al., 2020)	ER/AR binding; Aromatase inhibition; impairment of steroidogenesis; vitellogenin expression in response to xenoestrogen exposure; zona radiata, zona pellucida, DGE in er, bteb, tra, trb, thbzip,	Altered population sex ratio (Roco et al., 2021; Baranek et al., 2022); reduced fecundity

Endogenous activity associated with transitional phases of the anuran lifecycle, exogenous stressors that alter biological processes, methods to assess organismal effects, and potential demographic impacts. ↑ upregulation or increased expression; ↓ downregulation or decreased expression.
